# Dual echo bSSFP for real-time positive contrast of passive nitinol guidewires in MRI-guided cardiovascular interventions

**DOI:** 10.1186/1532-429X-16-S1-O79

**Published:** 2014-01-16

**Authors:** Adrienne E Campbell-Washburn, Toby Rogers, Michael Hansen, Robert J Lederman, Anthony Z Faranesh

**Affiliations:** 1 Division of Intramural Research, Cardiovascular and Pulmonary Branch, National Heart, Lung and Blood Institute, National Institutes of Health, Bethesda, Maryland, USA

## Background

MRI-guidance of cardiovascular catheterization procedures potentially offers an ionizing radiation-free alternative to X-Ray guidance. Imaging methods allowing visualization of commercially available passive guidewires would facilitate MRI-guided interventions. Previous work has demonstrated that positive contrast of paramagnetic materials, such as nitinol, can be generated with dephased echo sequences [1,2]. Here, we demonstrate a dual echo bSSFP sequence producing a wire image and an anatomical reference image in one TR, with variable flip angle for reduced SAR.

## Methods

**Figure 1:**
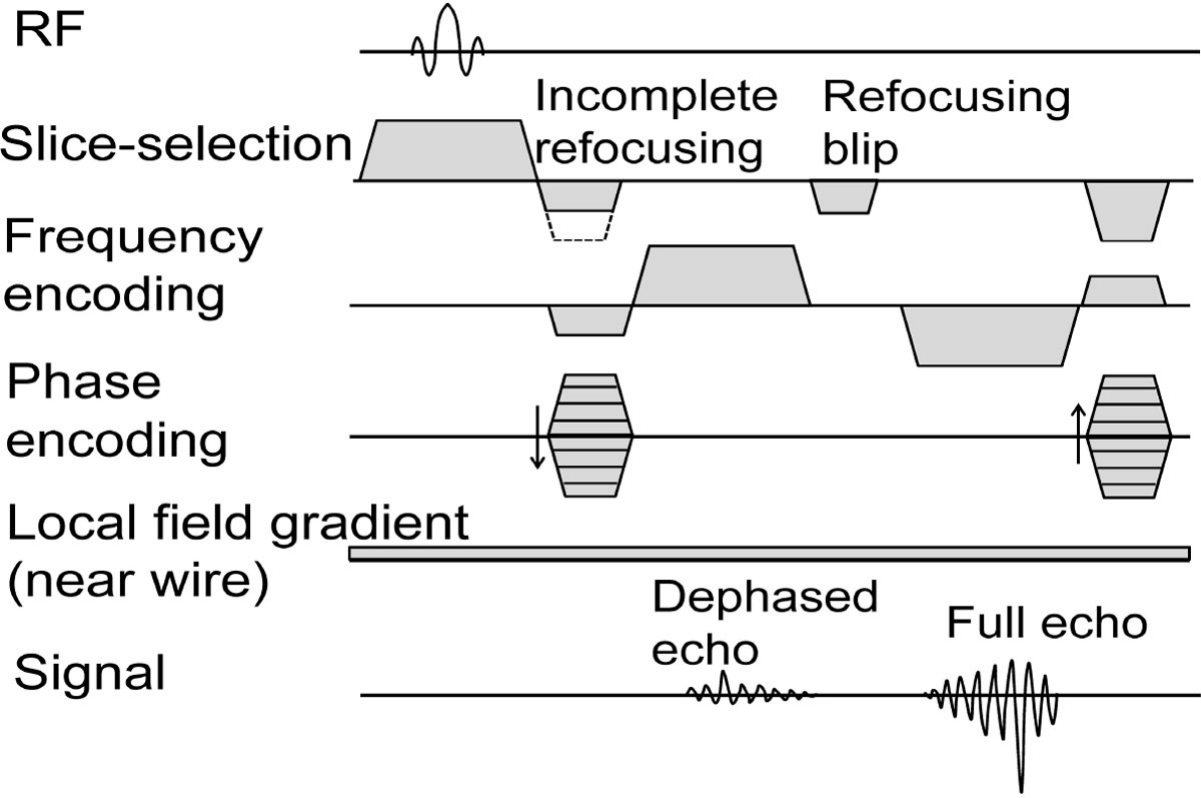
**Dual echo bSSFP pulse sequence diagram**.

The dual echo bSSFP sequence, uses incomplete slice-selective gradient refocusing to generate a dephased echo, exploiting local field gradients to complete refocusing and conserve signal around the wire only. Before the second echo, slice refocusing is completed to generate an anatomical reference image (Figure 1). Partial echoes (75%) were used to reduce TR. A variable flip angle scheme [3], with high flip angles only for center k-space lines, was implemented. Imaging was performed on a 1.5 T Siemens Aera scanner (Erlangen, Germany). Animal experiments were performed according to local ethical guidelines. A 0.035" Nitrex nitinol guidewire (Covidien, Dublin, Ireland) was inserted transfemorally to the descending thoracic aorta of a pig and imaged (TE_1_/TE_2_/TR = 1.15/2.83/3.90 ms, flip = 20-40°, 16 center lines acquired with high flip, 32 lines ramp flip up/down, matrix = 128^2^, slice thickness = 10 mm, slice refocusing gradient moment reduced by 50%).

## Results

**Figure 2:**
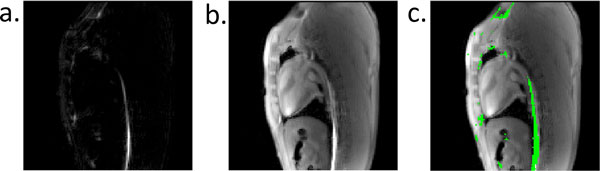
**a) Positive contrast wire image generated from dephased first echo, b) anatomical reference image generated from second echo and c) color overlay produced in MATLAB R2013a (Mathworks, Natick MA, USA) using a mean + 2 standard deviation signal intensity threshold from a)**.

The dephased echoes produce an image of the wire with positive contrast, which can be overlaid on the anatomical image for good visualization (Figure 2). Spurious additional signal comes from local field gradients at tissue interfaces. The variable flip angle scheme maintained good blood-myocardium contrast, while reducing the total RF energy to 51% compared to a constant flip angle of 40°. This sequence uses TR < 4 ms, generating both images in a 500 ms acquisition (fully sampled data).

## Conclusions

The application of this technique is limited by the wire itself, since the tapering of the nitinol shaft causes the tip to be less conspicuous. Future work will include testing of alternative wires. Parallel imaging will be used in future applications to reduce acquisition time and color overlay will be performed online. This method shows promise for improved visualization of commercially available guidewires with real-time MRI.

## Funding

This work was supported by the NHLBI DIR (Z01-HL006039-01, Z01-HL005062-08).
